# Draft Genome Sequence of *Oceanobacillus picturae* Heshi-B3, Isolated from Fermented Rice Bran in a Traditional Japanese Seafood Dish

**DOI:** 10.1128/genomeA.01621-15

**Published:** 2016-02-04

**Authors:** Sayuri Akuzawa, Junko Nagaoka, Motoki Kanekatsu, Yu Kanesaki, Tomonori Suzuki

**Affiliations:** a Department of Nutritional Science and Food Safety, Tokyo University of Agriculture, Tokyo, Japan; b Department of Biological Production, Tokyo University of Agriculture and Technology, Tokyo, Japan; c NODAI Genome Research Center, Tokyo University of Agriculture, Tokyo, Japan

## Abstract

*Oceanobacillus picturae* strain Heshi-B3 was isolated from rice bran in a traditional fermented seafood dish named Heshiko, which was produced in Fukui Prefecture in Japan. Here, we report the draft genome sequence of *O. picturae* strain Heshi B-3.

## GENOME ANNOUNCEMENT

Heshiko is one of the traditional fermented seafood dishes in Japan. It is produced by aging salted mackerel with rice bran for around 7 months. Some studies on fermented seafood have focused on the microbiological profile in rice bran and chemical changes in salted fish (1); however, they have not analyzed the genome sequence of the bacteria isolated from fermented rice bran. Here, we have newly isolated a bacterium belonging to the genus *Oceanobacillus* from Heshiko, and we deciphered the draft genome sequence. According to the similarity of genetic sequences, we named this strain *Oceanobacillus picturae* Heshi-B3.

Bacterial cells were cultured in medium (1% soluble starch, 0.5% yeast extract, 0.5% peptone, 0.1% K_2_HPO_4_, 0.02% MgSO_4_·7H_2_O, and 5% NaCl) at 30°C and were collected by centrifugation (10,000 × *g*, 15 min, 4°C). The cell pellet was treated with cell lysis solution (20 mg/ml lysozyme, 20 mM Tris-HCl [pH 8.0], 2 mM EDTA, 1.2% Triton X-100) for 30 min at 37°C. The bacterial genomic DNA was prepared using the DNeasy blood and tissue kit (Qiagen, Valencia, CA, USA). Five micrograms of genomic DNA dissolved in Tris-EDTA (TE) buffer was digested by a Covaris S-2 sonicator (Covaris, Woburn, MA, USA), with an average size of 500 bases. A DNA library was constructed using the NEBNext DNA library preparation kit (New England BioLabs, Ipswich, MA, USA), according to the manufacturer’s protocol. The library was sequenced by a massively parallel sequencer (MiSeq; Illumina KK, Tokyo, Japan) with a 300-base paired-end format, and finally, 2.90 Gb of sequence reads was obtained. The sequence reads were screened by a quality score higher than the Phred score of 30 and were each trimmed 10 bases from the both 5′ and 3′ ends. The trimmed reads were assembled *de novo* by CLC Genomics Workbench version 7.5 (Qiagen). Finally, the 92 contigs (>1 kb) were assembled, with a total length of 3,900,724 bp and *N*_50_ of 89,442 bp. Annotation of the contigs was performed by MiGAP (2). As a result, 9 rRNAs, 61 tRNAs, and 3,889 coding sequences (CDSs) were identified. So far, in the species of *O. picturae*, genomic sequences are available for only two strains, S1 (GenBank accession no. GCA_000612865.1) and Heshi-B3 (this work). Our sequencing data will largely contribute to future studies to understand the genetic variation among the species of *O. picturae*.

### Nucleotide sequence accession numbers.

Original sequence reads and metadata for this project were deposited to DDBJ/EMBL/GenBank under the accession numbers DRA003517 and PRJDB3832. Annotations and sequences of the assembled contigs are available under the accession numbers BBXV01000001 to BBXV01000092.
